# Nuclear Receptor Hepatocyte Nuclear Factor (HNF4) Controls Reproduction and Survival in Cotton Aphids by Regulating Lipid Metabolism

**DOI:** 10.3390/insects16121216

**Published:** 2025-11-28

**Authors:** Nanthini Sadasivam, Woo-Ram Park, Byungyoon Choi, Ji-Won Baek, Sunmin Kim, Hui-Jin Bae, Don-Kyu Kim

**Affiliations:** 1 Department of Integrative Food, Bioscience and Biotechnology, Chonnam National University, Gwangju 61186, Republic of Korea; 218545@jnu.ac.kr (N.S.); bjw237241@jnu.ac.kr (J.-W.B.); ksm6971@jnu.ac.kr (S.K.); hj210454@jnu.ac.kr (H.-J.B.); 2 Host-Directed Antiviral Research Centre, Chonnam National University, Gwangju 61186, Republic of Korea; wrpark@jnu.ac.kr (W.-R.P.); bychoi1035@jnu.ac.kr (B.C.)

**Keywords:** nuclear receptors, HNF4, cotton aphids, survival, reproduction, lipid metabolism, lipid mobilization

## Abstract

Nuclear receptors are a family of ligand-dependent transcription factors that regulate the development, reproduction, survival, and immune responses in insects. However, the structural, biological, and molecular functions of hepatocyte nuclear factor 4 (HNF4) in aphids remain unknown. Aphids are small agricultural pests that directly affect crops due to their rapid reproductive ability, which is influenced by environmental conditions such as temperature, humidity, and photoperiod. In this study, we cloned full-length cDNA encoding HNF4 from the cotton aphid *Aphis gossypii* (*A. gossypii*, Hemiptera: Aphididae) (*AgHNF4*) and investigated its role in regulating lipid metabolic gene expression, aphid survival, and reproduction. Phylogenetic analysis revealed that *AgHNF4* shares an evolutionary lineage with other hemipteran insects. The ligand binding domain region of *AgHNF4* showed significance in nuclear localization and transcriptional activity. The expression pattern of *AgHNF4* was detected in the stages of the aphid life cycle, with notable expression during the 2nd instar, 4th instar, and adult stages. Furthermore, to understand the role of HNF4 in regulating lipid metabolic genes involved in fatty acid synthesis and transport, HNF4-silenced *A. gossypii* individuals were analyzed. The results showed downregulation of lipid metabolic genes and reduced offspring production and survival rates. Therefore, *A. gossypii* HNF4 serves as a potential target for regulating lipid metabolism, and targeting HNF4 expression could offer a promising approach to aphid pest control.

## Simple Summary

Nuclear receptors are a family of ligand-dependent transcription factors that regulate key physiological processes, including development, reproduction, and metabolism in insects. Although nuclear receptors are known to be involved in several processes, the structural and functional role of hepatocyte nuclear factor 4 (HNF4) in aphids remain largely unexplored. Understanding how HNF4 contributes to lipid metabolism is essential to elucidate the molecular mechanism underlying aphid survival and reproduction. Therefore, this study aims to characterized the HNF4 gene in cotton aphid *Aphis gossypii* (*AgHNF4*), and investigate the functional role in lipid metabolism, survival, and reproduction though molecular characterization, expression profiling, and RNAi mediated gene silencing. These findings highlight *AgHNF4* as a potential molecular target for aphid pest management.

## 1. Introduction

The nuclear receptors (NRs) are ligand-dependent transcription factors that are constitutively expressed across diverse species [1,2]. NRs regulate gene expression and control various molecular and cellular processes [3]. Several NRs are considered orphan receptors because they lack endogenous ligands, although some are activated by small lipid-soluble molecules such as fatty acids, cholesterol, and steroid hormones [4,5]. The functions of NRs have been extensively studied in various animal groups and mammals to understand their roles in metabolism, homeostasis, and development [2,6,7,8,9]. Studies on insect NRs are emerging and are considered highly significant for understanding the role of insects in agricultural systems [10,11,12,13,14]. Hepatocyte nuclear factor 4 (HNF4) consists of two groups, HNF4α and HNF4γ, members of the nuclear receptor family that are expressed in the liver, kidney, pancreas, and intestine. These receptors are crucial regulators of liver-specific genes, primarily involved in lipid and glucose metabolism, contributing to cellular and molecular differentiation and development [15]. Dysregulation of HNF4 results in various disease conditions such as diabetes, hemophilia, cancer, inflammatory bowel syndrome, and hepatic steatosis. In humans, HNF4α contributes to lipid and glucose metabolism, and its dysfunction can alter triglyceride and plasma cholesterol levels, leading to lipid disorders [16]. HNF4 plays a significant role in lipid metabolism and homeostasis, particularly in regulating fatty acid oxidation, and has been reported to be involved in intestinal stem cell renewal and lipid catabolism through co-binding with acyl-CoA and fatty acyl-CoA thioesters [17,18]. Unlike humans, which have multiple HNF4 isoforms, insects have a single HNF4 ortholog [19]. The role of HNF4 in insects primarily relates to lipid metabolism, which is crucial for survival, reproduction, and development. *Drosophila melanogaster* (*D. melanogaster*) is considered a model insect for studying the link between lipid metabolism and development [20]. The role of lipids in *D. melanogaster* is vast, including regulating the conversion of lipid stores to very long-chain fatty acids and hydrocarbons that help maintain water homeostasis, enhancing life span and increasing desiccation resistance in adults. This, in turn, influences glucose levels and insulin resistance, indicating that HNF4 acts as a regulator of metabolic processes in adult flies [21]. Similarly, HNF4 in *D. melanogaster* is expressed in various tissues that are involved in lipid accumulation, storage, and transport. Lipid export and absorption have also been identified in enterocytes. Oenocyte (hepatocyte-like cells) interact with enterocytes to control and regulate intestinal lipid storage and export, and HNF4 has been reported to regulate this gut–liver axis [22]. In insects, lipid metabolism regulates oogenesis [19,23,24]. In *D. melanogaster*, HNF4 is reported to maintain lipid homeostasis and regulate oogenesis [19]. Knockdown of HNF4 in *Nilaparvata lugens* (*N. lugens*) results in reduced egg hatchability and significant changes in embryonic development, with HNF4 expression observed in fat bodies and ovaries regulated via ribosomal genes [25]. HNF4 silencing has been shown to partially block embryogenesis in the red flour beetle, *Tribolium castaneum* (*T. castaneum*) [10]. In the mosquito *Aedes aegypti* (*A. aegypti*), HNF4 is reported to regulate genes involved in triacylglycerol (TAG) degradation and β-oxidation, and its disruption results in an inability to metabolize lipids, leading to TAG accumulation [26]. HNF4 expression has also been observed in *Bemisia tabaci*, affecting desiccation tolerance and reproduction, along with silencing of vitellogenic genes [27]. With several notable previous studies on the role of HNF4 in other insects, here we focus on aphids.

Aphids are small, soft-bodied insects that primarily feed on plant phloem cells and simultaneously reproduce on their species-specific host plants [28,29]. Aphids have been studied for a long time; Anton van Leeuwenhoek in the 17th century was the first to report that aphids reproduce parthenogenetically through studies of aphid embryos. Later, Thomas Hunt Morgan in the early 20th century, while studying *Drosophila* genetics, also observed aphid cytogenetics to support the chromosome theory of sex determination. Thomas Henry Huxley in 19th century calculated that a single aphid can produce a biomass of offspring equivalent to that of 500 million men in ten generations, indicating the high fecundity of aphids, which is closely related to their regulatory mechanisms [30]. Aphids belong to the family Aphididae and the order Hemiptera and are major crop pests that adversely affect agricultural practices economically by transmitting plant viruses [31]. More than 5000 species of aphids have been identified worldwide, with 450 species associated with crop plants, and 100 species documented as major agricultural pests [32,33]. Ten aphid species are majorly associated with crop infestation worldwide: *Aphis gossypii* (*A. gossypii*) and *Acyrthosiphon gossypii* (cotton or melon aphids), *Lipaphis erysimi pesudobrassicae* (mustard or turnip aphids), *Myzus persicae* (*M. persicae*) (green peach aphids), *Sitobion avenae* (wheat aphids), *Acyrthosiphon pisum* (pea aphids), *Rhopalosiphum padi* (bird cherry-oat aphid), *Rhopalosiphum maidis* (corn leaf aphids), *Rhopalosiphum rufiabdominalis* (rice root aphids), and *Aphis glycines* (soybean aphids) [33].

Although studies have shown that HNF4 in insects is linked to lipid metabolism, storage, and transport, its mechanism of action in *A. gossypii* remains unknown. It is important to consider *A. gossypii* as a key model because it is a significant agricultural pest and a predominant plant virus vector, which paves way to study pest management, viral transmission in plants, and insect–plant interaction mostly distributed in the temperate and tropical regions [34,35]. *A. gossypii* is considered as a dangerous pest as it primarily feeds on economically important agricultural crops worldwide [36]. Thereby, there are over 50 plant viruses to crops and acts as a destructive pest [34]. The major action of *A. gossypii* is as a pest: it mainly sucks plant saps, infests buds and underneath the leaves, and cause significant damage resulting in retarded plant growth, malformed buds, and withered leaves [37]. Following plant sap ingestion, *A. gossypii* releases honeydew that promotes the formation of molds on the leaves [37]. Consequently, plant respiration and photosynthetic ability decline. If uncontrolled, *A. gossypii* may cause about a 44% reduction in agricultural crops by transmitting viruses [34], though there are many methods like insecticides and chemical pesticides to control *A. gossypii*. It is important to understand the molecular regulation and find a new approach that aims at a non-chemical and biological method for sustainable and effective aphid control. To address this gap, this study investigated the transcriptional activity of HNF4 in *A. gossypii,* which plays an important role in lipid regulation during reproduction and survival. We conducted molecular characterization of *AgHNF4*, RNAi silencing assays, and phenotypic analyses. *AgHNF4* RNAi silencing reduced HNF4 levels, which indirectly decreased the expression of lipid metabolic and transport genes. Phenotypically, we observed reduced survival rates and offspring production. These results confirm that *AgHNF4* is crucial for lipid metabolism and transport, which directly influences survival and reproduction.

## 2. Materials and Methods

### 2.1. Insect Rearing and Sample Collection

*A. gossypii* individuals were collected from a field collection in Gwangju (35°10′21″ N 126°53′57″ E) and is currently maintained at Chonnam National University, Republic of Korea. Aphids were reared on cucumber plants in growth chambers (laboratory-controlled conditions) at 25 ± 2 °C with a photoperiod of 16:8 h (light/dark) and a relative humidity of 60 ± 5%. Aphid population density and cucumber plant conditions were regularly monitored to prevent wilting and ensure insect survival. Adult aphids were carefully collected from the total population for RNA extraction and cDNA synthesis.

### 2.2. Sequence and Phylogenetic Analysis

*Aphis gossypii* HNF4 (*AgHNF4*) gene (Gene ID: 114123835) was obtained from the National Center for Biotechnology Information (NCBI) database using BLAST 2.17.0+, based on the amino acid sequence of *D. melanogaster* (Gene ID: 44544) (NP_723414.2). Sequence similarity and multiple protein sequence alignments were analyzed using Unipro UGENE version 51.0 (Unipro LLC, Akademgorodok, Novosibirsk, Russia), comparing *A. gossypii*, *D. melanogaster*, and *Homo sapiens* (*H. sapiens*). The evolutionary relationship of *AgHNF4* with other insect species, Diptera: Dm (*D. melanogaster*), Aa (*A. aegypti*), Coleoptera: Tc (*T. castaneum*), Ld (*Leptinotarsa decemlineata*), Lepidoptera: Bm (*Bombyx mori*, *B. mori*), Ms (*Manduta sexta*, *M. sexta*), Hymenoptera: Am (*Apis mellifera*), Cf (*Camponotus floridanus*), and Hemiptera: Ag (*A. gossypii*), Mp (*Myzus percisae*), Nl (*N. lugens*), was analyzed by constructing a phylogenetic trees using the neighbor-joining method with 1000 bootstrap replicates in Molecular Evolutionary Genetics Analysis (MEGA version 11.0.13). Amino acid sequences of *AgHNF4* and other insects were aligned using MUSCLE.

### 2.3. RNA Extraction and cDNA Synthesis

Total RNA was extracted from *A. gossypii* using Tri-RNA reagent (Favorgen Biotech Corporation, Ping-Tung, Taiwan) according to the manufacturer’s instructions. RNA concentration was determined using a Nano Drop spectrophotometer (Biophotometer D30, Eppendorf, Hamburg, Germany). First-strand cDNA was synthesized using 1 µg of total RNA using a cDNA synthesis Kit (TOPscript RT DryMIX Dt18 Plus; Enzynomics, Daejeon, Republic of Korea) according to the manufacturer’s instructions for gene cloning and real-time quantitative PCR (RT-qPCR) analyses. To analyze gene expression patterns during different developmental stages of the aphid life cycle, 1st, 2nd, 3rd, and 4th instar nymphs, as well as adult aphids, were carefully screened, and total RNA was extracted.

### 2.4. AgHNF4 Cloning, Plasmid, and DNA Construction

The full-length open reading frame (ORF) of *AgHNF4* was amplified using PCR from cDNA with specific primers targeting the 5′ and 3′ ends of the ORF and cloned into the pcDNA3/FLAG vector using EcoRV and XhoI restriction enzymes. The *AgHNF4* ligand binding domain (LBD) region (167–392 aa), excluding the activation function-1, DNA binding domain (DBD) region, and hinge domains, was subcloned into the pCMX/GAL-4 vector using EcoRV and NotI. Deletion constructs, *AgHNF4* dLBD (1–166 aa) and *AgHNF4* dH-LBD (1–148 aa) were subcloned into the pcDNA3/FLAG vector using EcoRV and NotI. 8×-HNF4-RE-luc contained 8 copies of the HNF4 binding site, and pFR-luc contained 5 copies of the GAL4 binding site were previously described [38,39]. Primers used for cloning are listed in Table S1. All cloned plasmids were validated by DNA sequencing (Bionics, Seoul, Republic of Korea)

### 2.5. Cell Culture and Transient Transfection Assay

293T cells were cultured in Dulbecco’s Modified Eagle’s Medium (DMEM, high glucose; Welgene, Gyeongsan-si, Republic of Korea) and maintained at 37 °C with 5% CO_2_. Cells were passaged and monitored at regular intervals at room temperature. Transient transfection was performed to analyze gene expression and transcriptional activity. pcDNA3/FLAG/*AgHNF4*, pCMX/GAL4/*AgHNF4*-LBD, pcDNA3/FLAG/*AgHNF4* dLBD, and pcDNA3/FLAG *AgHNF4* dH-LBD constructs were transfected using polyethyleneimine (PEI; Polysciences Inc., Warrington, PA, USA) based on the manufacturer’s instructions. The total DNA concentration was adjusted to 1 µg per well, with an empty vector used as a control. The Nano-Glo vector was used as an internal control, and firefly luciferase activity was normalized to Nano-Glo luciferase activity. All experiments were performed in triplicate.

### 2.6. Quantitative PCR Assay

The gene expression pattern of *AgHNF4* was analyzed by RT-qPCR. Total RNA was extracted at different developmental stages of *A. gossypii* and reverse transcribed into cDNA using TOPscript RT DryMIX (Enzynomics). RT-qPCR was performed on a CFX Connect Real-Time System (Bio-Rad, Hercules, CA, USA). Beta-actin (β-actin) and elongation factor-1 alpha (EF1α) primers validated for *A. gossypii* were used to normalize mRNA expression levels. *β-Actin* was used to normalize *AgHNF4*, whereas *EF1α* was used as a normalization for fatty acid synthase (FAS), acetyl Co A carboxylase (ACC), sterol regulatory element binding protein (*SREBP*), lipophorin receptor (LpR), apolipophorin (ApoLpp). *β-Actin*, and *EF1α* were designed using Primer-BLAST along with their accession number (Table S1). Relative gene expression was calculated using the ∆∆Ct method.

### 2.7. dsRNA Synthesis and Injection

Primers for *AgHNF4* and pEGFP-N1 containing a T7 promoter sequence at the 5′ end were designed (Table S1). Double-stranded RNA (dsRNA) for *AgHNF4* (298 bp) and pEGFP-N1 (392 bp) were amplified through PCR using the respective primers, and fragment sizes were confirmed by gel electrophoresis. The dsRNAs (*dsHNF4 and dsGFP*) were synthesized using Ampliscrible T7-Flash Transcription Kit (Epicenter Technologies, Madison, WI, USA) according to the manufacturer’s instructions. Synthesized dsRNA was purified and dissolved in RNase-free water. The final concentration of the synthesized dsRNA was measured using a Biophotometer D30 (Eppendorf, Hamburg, Germany). The knockdown effects of HNF4 expression on aphids were observed in the adult stages. Adult aphids were injected with *dsGFP* (*n* = 100; 23 nL of 4.5 µg/µL) and *dsHNF4* (*n* = 100, 23 nL injection of 4.5 µg/µL). Injections were administered at the midpoint of the aphid abdomen using a micro-dispenser (VWR Scientific, Radnor Township, PA, USA) fitted with a polished glass pipette (20 nm in diameter). After injection, RNA was extracted from the pooled samples of 100 aphids (*dsGFP*, *n* = 100; *dsHNF4*, *n* = 100). Fresh aphids were used for extraction weighing around 30–60 mg per injection group using 1 mL of Tri-RNA reagent (Favorgen Biotech Corporation, Ping-Tung, Taiwan) according to the manufacturer’s instructions.

### 2.8. Western Blot Assay

Proteins were extracted from 293T cells transfected with pcDNA3/FLAG/*AgHNF4* using PEI (Polysciences, Inc., Warrington, PA, USA) according to the manufacturer’s instructions. Whole-cell protein extracts were prepared using RIPA buffer (Thermo Fisher Scientific, Rockford, IL, USA) as previously described [39]. Following centrifugation at 16,000 rpm for 15 min at 4 °C, the supernatant was transferred to fresh tubes, and total protein concentration was quantified using the Bradford assay. A total of 100 µg of protein was loaded onto a 10% SDS-PAGE gel and transferred to nitrocellulose or polyvinylidene fluoride membranes (EMD, Millipore, Danvers, MA, USA). The membrane was blocked in 5% skim milk dissolved in TBST for 1 h at RT. The membrane was then probed with primary antibodies, anti-FLAG tagged (1:1000) (monoclonal mouse antibody) and Actin-antibody (monoclonal mouse antibody) (1:1000) (Santa Cruz Biotechnology, Dallas, TX, USA). Following this, membranes were incubated with horse radish-peroxidase conjugated goat anti-mouse IgG secondary antibody (1:3000 dilutions, Bethyl, Montgomery, AL, USA). The membranes were then incubated in ECL reagent (GE Healthcare, Arlington Heights, IL, USA) for 1 min. Protein bands were visualized using the ChemiDoc XRS imaging system (Bio-Rad Laboratories, Hercules, CA, USA). Nuclear and cytosolic proteins were also extracted using the Nuclear/Cytosol Fractionation Kit (BioVision, Milpitas, CA, USA) from 293T cells transfected with FLAG-*AgHNF4* WT and FLAG-*AgHNF4* dH-LBD using PEI (Polysciences, Inc., Warrington, PA, USA), following the aforementioned protocol. Membranes were probed with primary antibodies: anti-FLAG tagged (1:1000), anti-Lamin A/C (1:1000), and anti-tubulin (1:1000) (Santa Cruz Biotechnology, Dallas, TX, USA), and treated with horse radish-peroxidase conjugated goat anti-mouse IgG secondary antibodies (1:3000, Bethyl, Montgomery, AL, USA). The membranes were incubated in ECL, and protein bands were visualized for nuclear and cytosolic protein expression.

### 2.9. Immunocytochemistry

293T cells were cultured on chamber slides (Lab-Tek II Chamber slide system, Thermo Fisher Scientific) and transfected with FLAG-*AgHNF4* WT and FLAG-*AgHNF4* dH-LBD using PEI (Polysciences, Inc.). Immunofluorescence staining was performed using a DYKDDDDK-tag monoclonal antibody (1:1000 dilution) followed by Alexa-Fluor 488 conjugated goat anti-mouse secondary antibody (1:1000 dilution). DAPI (4′,6-diamidino-2-phenylindole) was added to the cells, and the samples were mounted on a glass slide. Images were captured using Steve full v1.6.3496 software.

### 2.10. Survival and Offspring Production Assay

Adult aphids were carefully collected and divided into two groups, *dsGFP* (control) and *dsHNF4* (target). Each group consisted of two replicates, with *n* = 10 aphids per replicate. Survival was monitored daily from day 1 to day 3 to record mortality. Adult aphids were injected with *dsGFP* or *dsHNF4* (*n* = 10 aphids per replicate, total of 2 replicates). Offspring production was measured 3 days post-injection to minimize external stress that could affect reproduction. The number of offspring produced by each individual aphid injected from both replicate groups was recorded.

### 2.11. Aphid Dissection

Adult aphids were collected for dissection. The collected aphids were placed in a cold phosphate-buffered saline (PBS) solution in a Petri dish for 5 min on ice to immobilize them. Subsequently, aphids were placed in a Petri dish containing solidified agar medium for stabilization. The ventral side was positioned upward, and the aphids were held with tweezers at both ends. The head was carefully separated from the thorax and abdomen. The cuticle was gently cut open, and the ovaries were removed. The dissected ovaries or samples were placed in cold PBS in fresh tubes for further analysis.

### 2.12. Oil Red O Staining

*A. gossypii* ovaries were dissected in PBS and fixed in 4% formaldehyde for 10–20 min at RT. The samples were rinsed with distilled water and incubated in Oil Red O solution (Sigma-Aldrich, St. Louis, MO, USA, O0625-100G) for 45 min at RT. After staining, the samples were rinsed again with distilled water. Lipid droplets, visualized as red-stained regions, were quantified using ImageJ 1.54g (Rasband, W.S, ImageJ, U.S. National Institute of Health, Bethesda, MD, USA).

### 2.13. Statistical Analysis

Statistical analyses were performed using GraphPad Prism (GraphPad software, Version 5.01, La Jolla, CA, USA). All data were presented as the mean ± SD. Significance was determined using a two-tailed Student’s *t*-test. Differences were considered statistically significant at *p* ˂ 0.05.

## 3. Results

### 3.1. Sequence and Structural Analysis of AgHNF4

**Figure 1:**
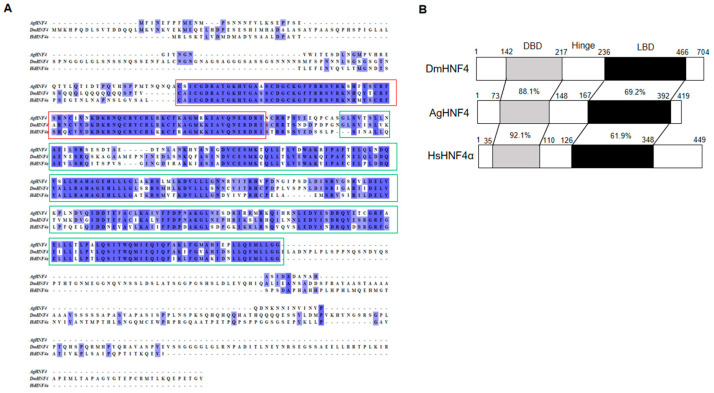
Sequence alignment and domain structure of *AgHNF4*. (**A**) Multiple sequence alignment of HNF4 proteins from *DmHNF4*, *AgHNF4*, and *HsHNF4α*. Conserved regions are shaded in blue, with DBD (red box) and LBD (green boxes) highlighted, showing high sequence conservation across species. (**B**) Schematic representation of HNF4 amino acid domain structures. The DBD, hinge region, and LBD are indicated. Percentages indicate the amino acid identity between *AgHNF4* and its orthologs *DmHNF4* and *HsHNF4α*, demonstrating strong conservation in the DBD and moderate conservation in the LBD.

**Figure 2:**
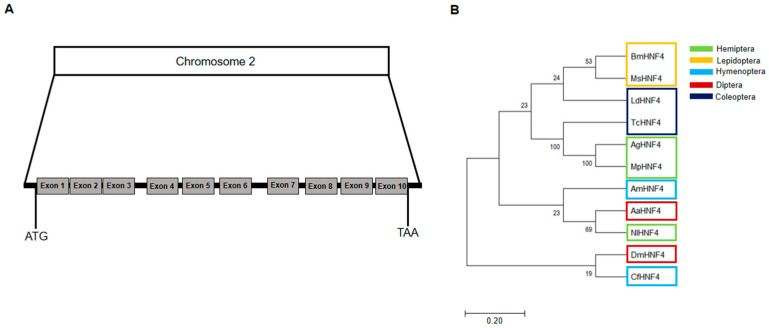
*AgHNF4* identification and phylogenetic tree analysis. (**A**) Genomic organization of the gene located on chromosome 2, consisting of 10 exons spanning from the ATG start codon to the TAA stop codon. (**B**) Phylogenetic tree analysis of HNF4 proteins from different insect orders (Hemiptera, Lepidoptera, Hymenoptera, Diptera, Coleoptera). *AgHNF4* clusters with other hemipteran HNF4 proteins, indicating evolutionary conservation. The accession numbers and abbreviations for HNF4 for different species are provided in the Section 2.

The *AgHNF4* sequence was obtained from NCBI and compared to *D. melanogaster* HNF4 (*DmHNF4*) using BLAST, revealing four isoforms XP_027842742.2 (420 aa), XP_027842743.2 (419 aa), XP_027842744.2 (384 aa), XP_027842745.2 (376 aa), with 72% similarity. Among these, XP_027842743.2 (419 aa) was successfully cloned and used as the query sequence for multiple sequence alignment and phylogenetic tree construction. Multiple sequence alignment between *AgHNF4*, *DmHNF4*, and *H. sapiens* HNF4 alpha (*HsHNF4α*) showed a conserved DBD region and LBD region (Figure 1A). Furthermore, functional domain analysis of *AgHNF4* showed that the gene encodes a protein sharing 88.1% and 92.1% similarity in DBD region and 69.2% and 61.9% similarity in the LBD region with *DmHNF4* and *HsHNF4α*, respectively (Figure 1B). Additionally, HNF4 sequences are highly conserved in insects. The genomic structure of *AgHNF4* is mapped to chromosome 2, consisting of 10 exons from the start codon (ATG) to the stop codon (TAA), indicating a conserved genomic structure (Figure 2A). Phylogenetic tree analysis demonstrated the evolutionary relationship between *A. gossypii* HNF4 and other insect species (Figure 2B). *M. persicae* (Hemiptera) and *T. castaneum* (Coleoptera) were more closely related to *A. gossypii* HNF4, as expected. These results suggest that *AgHNF4* belongs to the same evolutionary branch as other hemipterans.

### 3.2. AgHNF4 Positively Regulates Basal Transcriptional Activity

**Figure 3:**
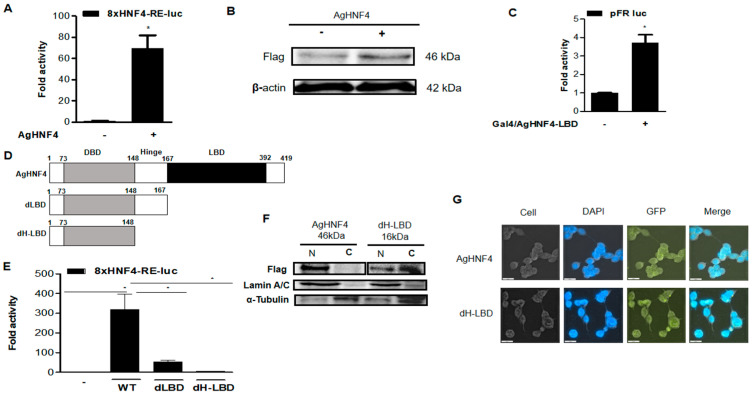
*AgHNF4* transcription level analysis. (**A**) Luciferase reporter assay showing that *AgHNF4* significantly activates the 8×HNF4-RE-luc reporter. (**B**) Western blot analysis showing the expression of *AgHNF4* in FLAG-tagged samples compared with the control (empty vector). The expected protein band was detected at 46 kDa, with β-actin (42 kDa) used as a loading control. (**C**) Gal4-based reporter assay demonstrating that LBD of *AgHNF4* possesses transcriptional activity. (**D**) Schematic representation of full-length *AgHNF4* and deletion constructs lacking the dLBD and dH-LBD regions. (**E**) Reporter assays comparing transcriptional activity of wild-type *AgHNF4* (WT) and deletion mutants (dLBD, dH-LBD) on the 8×HNF4-RE-luc reporter. Loss of the LBD dramatically reduced transcriptional activation. (**F**) Subcellular fractionation shows that *AgHNF4* localizes predominantly in the nucleus, whereas the dH-LBD mutant displays partial localization in nucleus and predominantly in cytoplasm. Lamin A/C and α-tubulin served as nuclear and cytoplasmic markers, respectively. (**G**) Immunofluorescence microscopy confirmed nuclear localization of *AgHNF4*, whereas partial localization in nucleus and strong signals in cytoplasm of dH-LBD. The fluorescence was recorded and observed in cells, DAPI stain filter, GFP filter, and merged (DAPI and GFP) images to differentiate the localization. Scale bars = 20 µm. Luciferase activity in panels (**A**,**C**,**E**) was measured after 48 h of transfection. Data are presented as the mean ± SD. * *p* ˂ 0.005 using a two-tailed Student’s *t*-test.

The transcriptional activity of the *AgHNF4* construct was analyzed using the 8×HNF4 RE-Luc reporter construct, which was transfected into 293T cells, as *AgHNF4* shares a conserved DBD domain with mammals. As expected, *AgHNF4* activity significantly increased, and expression levels remained stable (Figure 3A). Consistent with these findings, *AgHNF4* protein levels were also analyzed, showing increased band intensity in *AgHNF4* transfected cells (+) compared to the control transfected with empty vector (−) (Figure 3B). To study the transcriptional role of the *AgHNF4* LBD region, *AgHNF4*-LBD was transfected into 293T cells using pFR-Luc as a reporter construct. As expected, the experimental results showed a significant increase in folding activity of *AgHNF4*-LBD, suggesting that LBD is crucial for recruiting coactivators and ligands to stimulate transcriptional activity (Figure 3C). These results demonstrate that *AgHNF4* is a transcriptionally active nuclear receptor, with both its DBD and LBD contributing to its regulatory function.

### 3.3. AgHNF4 LBD Is Required for Nuclear Localization

Deletion constructs of *AgHNF4* dLBD and *AgHNF4* dH-LBD were transfected with 8×HNF4-RE-luc into 293T cells to analyze the role of LBD in nuclear localization. First, schematically, the deletion constructs (*AgHNF4* dLBD and *AgHNF4* dH-LBD) were compared with wild-type *AgHNF4* (Figure 3D). Later, the transcription levels via reporter assay were analyzed after 48 h. As expected, the transcriptional levels of dLBD and dH-LBD were significantly decreased, indicating that the LBD is crucial for the active transcription of *AgHNF4* (Figure 3E). To further support these findings suggesting the crucial role of LBD, we investigated the protein levels by transfecting with 8×HNF4-RE into 293T cells to analyze the nuclear and cytosolic localization of *AgHNF4* and dH-LBD via Western blot. As expected, the Western blot analysis showed bands more intense in the nuclear fraction of *AgHNF4*, whereas dH-LBD was predominantly expressed in the cytoplasmic fraction and with low band intensity in the nuclear fraction (Figure 3F). Consistent with these results, the subcellular localization analyzed through immunofluorescence staining using the FLAG-specific monoclonal antibody, followed by the Alexa Fluor 488 conjugated secondary antibody and nuclei counterstained with DAPI of deletion construct (dH-LBD) and *AgHNF4* in vitro were analyzed. The results showed that the dH-LBD alters the subcellular localization within the cells (Figure 3G). These data confirm that *AgHNF4* localizes to the nucleus and that this localization depends on the sequence containing the LBD.

### 3.4. HNF4 Gene Expression Is Crucial in Developmental Stages of Aphids

**Figure 4:**
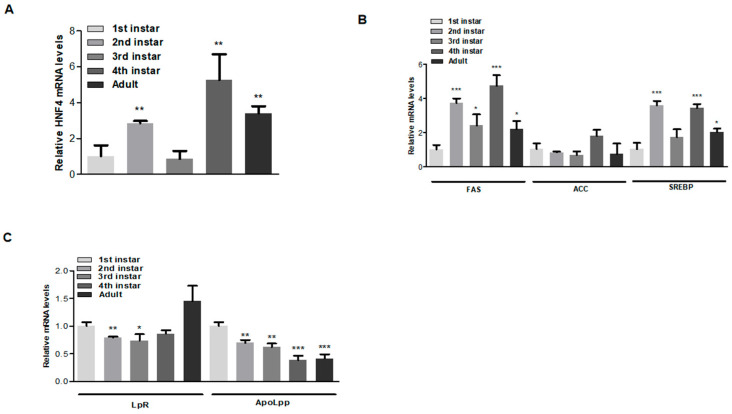
*AgHNF4* regulates lipid metabolic and transport gene expression. (**A**) Relative *AgHNF4* mRNA expression levels at different developmental stages (1st instar to adult) determined by RT-qPCR analysis. Expression levels were significantly higher in the 2nd instar, 4th instar, and adult stages (** *p* < 0.01). (**B**,**C**) Expression patterns of lipid metabolic and transport genes during aphid developmental stages analyzed via RT-qPCR. The expression patterns were similarly significant to those of *AgHNF4.* Data presented as the means ± SD (* *p* ˂ 0.05, ** *p* ˂ 0.01, *** *p* ˂ 0.001, using two-tailed Student’s *t*-test.

To examine HNF4 expression levels in *A. gossypii* during various developmental stages, mRNA expression was analyzed across stages from the 1st instar to adult. Expression levels were significantly higher in the 2nd instar, 4th instar, and adult stages (Figure 4A). Similarly, genes involved in lipid metabolism and lipid transport were analyzed to assess their effects during aphid development. Notably, FAS and *SREBP* showed a similar expression pattern to HNF4, whereas ACC was not significantly affected (Figure 4B). For LpR and ApoLpp, LpR mRNA levels were consistent and significantly higher during the 2nd and 3rd instar stages, peaking at the adult stage. ApoLpp levels gradually decreased but remained significant across developmental stages (Figure 4C). We speculate that ApoLpp, being a lipid transport protein, circulates throughout development and declines during the adult stage. These findings suggest that HNF4 expressions are developmentally regulated and play a predominant role in specific stages of aphid development.

### 3.5. AgHNF4 RNAi Silencing Affects Lipid Metabolic Gene Expression and Reduces Survival and Offspring Production in Cotton Aphids

**Figure 5:**
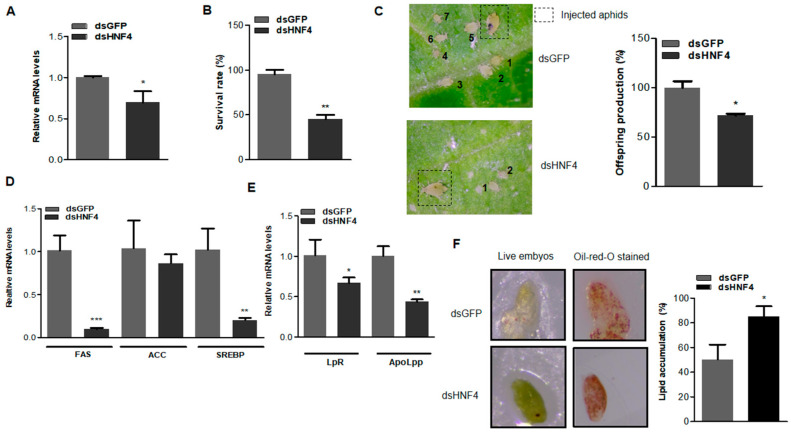
RNAi mediated *AgHNF4* negatively affects offspring and survival. (**A**) Aphids injected with *dsGFP* and *dsHNF4* (*n* = 100 per injection group) were isolated on day 3 post-injection. Total RNA was isolated and analyzed using RT-qPCR. *AgHNF4* knockdown showed significant downregulation of relative mRNA levels. (**B**) Survival rate of adult aphids injected (*n* = 10 per replicate, total 2 replicates) with *dsGFP* and *dsHNF4* was measured 3 days post-injection. (**C**) Offspring production following RNAi-mediated HNF4 knockdown. Aphids injected with *dsGFP* (*n* = 10 per replicate, total 2 replicates) and *dsHNF4* (*n* = 10 per replicate, total 2 replicates) showed a significant reduction in offspring count (the image represents a random individual aphid from the replicates and the offspring count). Offspring were recorded and labeled in numbers according to their respective groups. (**D**,**E**) Total RNA isolated from *dsGFP*- and *dsHNF4*-injected aphids analyzed for mRNA levels of lipid metabolic and transport genes. (**F**) Dissected aphid embryos stained with Oil Red O. Three days post-RNAi injection with *dsGFP* and *dsHNF4*. Quantification of lipid accumulation (%) shows a significant increase in lipid accumulation in *dsHNF4* injected aphids compared to controls. Data are presented as the means ± SD. (* *p* ˂ 0.05, ** *p* ˂ 0.01, *** *p* ˂ 0.001, two-tailed Student’s *t*-test).

*AgHNF4* transcript levels in aphids treated with *dsHNF4* were significantly reduced compared to the *dsGFP* control. This statistically significant decrease in mRNA levels confirms efficient *AgHNF4* silencing by RNAi, thereby validating the knockdown approach for functional and phenotypic characterization (Figure 5A). To examine the effects of HNF4 knockdown on aphid survival, aphids injected with *dsGFP* (control) and *dsHNF4* were compared. Aphids injected with *dsGFP* appeared active and healthy, with a survival rate of approximately 95%. In contrast, *dsHNF4* treated aphids showed a significantly reduced survival rate of 45% (Figure 5B). These observations confirm that HNF4 is a crucial transcription factor involved in the survival of *A. gossypii*.

Offspring production was also analyzed to assess the impact of HNF4 regulation on reproduction. Aphids injected with *dsGFP* exhibited normal offspring production, whereas *dsHNF4*-mediated knockdown resulted in significantly reduced offspring counts, indicating the involvement of HNF4 in reproduction (Figure 5C). To further assess whether lipid metabolic and transport genes were affected by HNF4 knockdown, the mRNA levels of FAS, ACC, *SREBP*, LpR, and ApoLpp were analyzed. As expected, mRNA levels of these genes were significantly reduced, confirming that HNF4 likely regulates downstream lipid metabolic and transport genes influencing survival and offspring production (Figure 5D,E). Collectively, these results indicate that HNF4 plays an essential role in regulating lipid mobilization and metabolism. To further assess the physiological effects, Oil Red O staining was performed to visualize lipid accumulation. Five individual aphids were collected simultaneously after offspring count from respective injected groups and dissected. *dsGFP*-injected aphids showed reduced lipid staining, indicating normal fat storage and mobilization throughout development and reproduction. In contrast, *dsHNF4*-injected aphids showed stronger staining; quantification of lipid droplet accumulation confirmed this observation. The total lipid area was higher in *dsHNF4* compared with *dsGFP*-injected aphids (Figure 5F). These results suggest that HNF4 knockdown disrupts lipid mobilization and facilitates lipid accumulation.

## 4. Discussion

HNF4 is a well-known nuclear receptor that regulates various biological activities across different species. In this study, we first identified and isolated the full-length HNF4 cDNA coding gene in cotton aphids. We then cloned HNF4 and characterized its function in lipid metabolism and lipid mobilization. The amino acid sequence of *AgHNF4* within the DBD showed 88.1% and 92.1% similarity with *DmHNF4* and *HsHNF4α*, respectively. The LBD showed 69.2% and 61.9% similarity. Analysis of the amino acid sequence revealed that the DBD was significantly conserved, whereas the LBD was less conserved across species. Interestingly, the AF-2 region was significantly less conserved in *AgHNF4*. Phylogenetic analysis showed that HNF4 sequences clustered according to insect orders. The *AgHNF4* sequence clustered closely with other hemipterans, such as *MpHNF4* (green peach aphid), and branched out near the coleopteran insect *TcHNF4* in the phylogenetic tree. We speculate that *A. gossypii*, *M. persicae*, and *T. castaneum* HNF4 are homologous genes that are evolutionarily conserved and may have similar biological functions. Furthermore, we found that *AgHNF4* is located on chromosome 2 and contains 11 exons. This observation provides structural insight into its transcriptional and translational mechanisms, which confirms that exon splicing is critical for functional protein translation. To comprehensively understand the function of HNF4 expression in cotton aphids, we analyzed mRNA levels across developmental stages from the 1st instar to adult; protein levels were also analyzed through Western blot analysis. The results showed consistent HNF4 expression level throughout development, suggesting its important role in *A. gossypii*. Development is an important biological process across all organisms, particularly in insects [40,41]. Hemimetabolous insects such as aphids grow, mature, and reproduce throughout the nymphal stages [42]. The mRNA expression levels observed in this study support this pattern.

It is well established that proper development and reproduction are essential for insects. These cellular processes are controlled through metabolic processes and lipid transport between the midgut, fat body, and ovaries [43]. Metabolism continuously regulates and coordinates development and reproduction throughout the life cycle of insects. Reports on brown planthoppers have shown that the downregulation of glycolytic enzymes reduces fecundity [44,45], suggesting the involvement of glycolytic metabolic genes in reproduction. Similarly, in green peach aphids, glycolytic genes have been shown to regulate fecundity [46]. More specifically, lipid metabolism and its associated genes have been shown to be crucial to reproduction, development, and survival [19,47]. Developmental expression profiling demonstrated that *AgHNF4* mRNA levels were consistently expressed from the first instar to the adult stage, with higher expression during the 2nd instar, 4th instar, and adult stages. This pattern suggests a predominant role for HNF4 in crucial stages of development. We speculate that HNF4, as a transcription factor, may act as an upstream regulator controlling lipid metabolic genes (FAS, ACC, *SREBP*) and lipid transport genes (LpR and ApoLpp). In the results, these genes were shown to be differentially expressed; however, their biological functions were not elaborated. To fully interpret these results, it is essential to discuss the potential roles of each gene involved in insects’ lipid metabolism. In insects, FAS, ACC, and *SREBP* genes are considered the central components of the lipid metabolism pathway. FAS is the key multiple enzyme coordinates along with ACC and also the target for *SREBP* to synthesize fatty acid [48,49]. FAS is involved in various physiological and cellular functions in insects from metabolism, energy storage by accumulating triglycerides, acting as a major structural component for the cells, and also involves in insects’ cuticular formations, development, and reproduction process and supports the immune response in certain arboviral infections in mosquitoes by acting as a membrane component essential for viral replications [50,51,52,53,54]. Similarly, ACC is a key enzyme involved in the *de novo* fatty acids synthesis pathway that acts as a *SREBP* direct target like FAS [55,56]. During fatty acid synthesis, ACC catalyzes the lipid synthesis by converting the acetyl-CoA to malonyl-CoA, the major regulatory point in lipid synthesis pathway [57]. Also, physiologically, ACC is critical for reproduction; deficiency in ACC leads to fewer egg production and altered eggshell structures reported in blood feeding bug, *Rhodnius prolixus* [58]. The function of ACC is not limited to reproductive processes but also plays an important role in nutrient homeostasis involved in digestion and fat body lipogenesis and TAG transport and lipid storage [59]. Together, FAS and ACC form the essential mechanism that drives insects’ lipid synthesis while *SREBP* majorly functions as the transcriptional factor that regulates FAS and ACC [60,61]. Insects synthesize lipid *de novo* and regulate it via diet [61]. Thus, *SREBP* controls fatty acids, triglycerides, and phospholipids. Reports have shown that *SREBP* is essential for larval development and survival in *D. melanogaster*. *SREBP* knockdown larvae shows a mortality at the second instar stage unless provided with dietary lipids [62,63]. Also, most importantly, dysregulation of *SREBP* results in excessive lipid accumulation and metabolic imbalance [64]. The lipid synthesis via FAS, ACC, and *SREBP*-mediated pathways are further distributed and recycled when LpR—a low density lipoprotein receptor found in midgut, fat body, brain and ovaries—mediates the uptake of diacylglycerol from high-density lipophorin particles, thereby supplying energy to demanding tissues during development and reproduction [65]. Unlike mammalian LpR, insects’ LpR act as a reusable shuttle mechanism, recycling both the receptors and lipophorin to the cell surface for lipid transport [66]. The LpR isoform, *LpR1*, demonstrates its role in the *D. melanogaster* nervous system, which facilitates the neuroglia lipid shuttle and dendritic morphogenesis [67,68] whereas ApoLpp synthesized in the fat body form the structural and exchangeable protein components of lipophorin particles. ApoLpp has three isoforms: ApoLpp I/II/III [69]. ApoLpp I/II contribute to the cuticular hydrocarbon transport involved in chemical communication and waterproofing [70]. ApoLpp III is associated with lipid mobilization and innate immunity by stimulating antimicrobial peptide production [71]. Taken together, these genes including FAS, ACC, *SREBP*, LpR, and ApoLpp comprise an interconnected lipid regulatory network that regulates lipid synthesis, transport, storage, and utilization in insects. Considering the relationships, our analysis showed that FAS and *SREBP* showed expression patterns similar to HNF4 across developmental stages, whereas LpR is expressed throughout all developmental stages in aphids. However, ApoLpp, the major lipid transporter carrying TAG and diacylglycerol, is distributed during early development and declines as aphids reach the adult stage. Similar findings have been reported in *M. sexta* [72]. Additionally, similar developmental regulation of HNF4 has been reported in *D. melanogaster* and *N. lugens*, where HNF4 regulates lipid homeostasis and metabolism [19,21,22,25,47], highlighting the essential role of HNF4 and its downstream lipid metabolic and transport genes.

RNAi-mediated silencing of *AgHNF4* significantly reduced transcript levels, confirming efficient knockdown. Functionally, HNF4-silenced aphids exhibited delayed development, decreased survival, and reduced reproduction rates. These findings align with previous reports in *T. castaneum* and *A. aegypti*, where HNF4 depletion impaired lipid metabolism, embryogenesis, and survival [26,73]. In this study, mRNA analysis of *dsHNF4*-treated aphids revealed a reduced expression of lipid transport and metabolic genes. This suggests that these genes are regulated by HNF4. Previously, HNF4 was reported to regulate lipid metabolic and transport genes in *D. melanogaster* [22]. Our study confirms that *AgHNF4* is necessary for proper lipid transport, utilization, and metabolism, linking transcriptional regulation to physiological functional outcomes.

To further support this hypothesis, we observed a reduction in reproductive output and survival in HNF4-silenced aphids, two of the most important phenotypic outcomes of impaired lipid mobilization and metabolism. Aphids rely on lipid reserves stored in the fat body for embryo development, and impaired lipid metabolism likely restricts nutrient availability to developing embryos, resulting in reduced fecundity. Similar findings have been reported in other insects such as *B. mori* [74]. These results highlight HNF4 as a central coordinator of metabolic and transport genes that might be responsible for reproductive and survival processes in aphids.

Overall, our study demonstrates that *AgHNF4* is a key transcriptional regulator of lipid metabolism, mobilization, survival, and reproduction in *A. gossypii*. Targeting HNF4 could represent a novel strategy for aphid pest control by disrupting lipid homeostasis and reducing survival and reproductive capacity. At present, no studies have reported the development of *AgHNF4*-specific inhibitors in insects. However, in mammalian HNF4, several small molecules such as BI6015, Alverine, Benfluorex, and fatty acids (e.g., linoleic acid) have been identified as modulators or inhibitors of HNF4α [75,76,77,78]. Although there is no direct evidence demonstrating the inhibition of insect or *AgHNF4* activity by these compounds, they could serve as potential lead molecules for future screening and characterization studies aimed at understanding and regulating *AgHNF4* function in aphids. Also, future studies should focus on identifying specific downstream target genes of *AgHNF4* that regulate physiological activities in *A. gossypii* and on elucidating the molecular mechanisms linking lipid regulation to reproduction in aphids.

## Data Availability

The original contributions presented in this study are included in the article/Supplementary Materials. Further inquiries can be directed to the corresponding author.

## Supplementary Materials

The following supporting information can be downloaded at: https://www.mdpi.com/article/10.3390/insects16121216/s1, Table S1: Primer list.
