# Supplementary material for: Nuclear Receptor Hepatocyte Nuclear Factor (HNF4) Controls Reproduction and Survival in Cotton Aphids by Regulating Lipid Metabolism
Source: Insects. 2025 Nov 28;16(12):1216. doi: 10.3390/insects16121216 (PMC12733786; doi:10.3390/insects16121216)
Supplement: Supplementary file 1 [file insects-16-01216-s001.zip › insects-3971387-supplementary.pdf]

Table S1. Primer sequences

Primer	Accession Number	Primer sequences (5'-3')
AgHNF4	XM_027986942.2	Forward<br>ATGTTCATAAACGAATTTCCATTCATGGAG<br>Reverse<br>TTATGGCACGTTGATGACATTGATGTTGTT
*q-AgHN4	XM_027986942.2	Forward<br>CGTATGGATAACAGAATCGGACCTCAACGG<br>Reverse<br>CTTGATTCTGGTTGGTCATGGGCGGCGAGT
AgHNF4-LBD	XM_027986942.2	Forward<br>TCCGTCACATCACTATTGAACGCGGAAATC<br>Reverse<br>CATCTCCTGTAACAACGGTTCGATGTGTGC
AgHNF4-dLBD	XM_027986942.2	Forward<br>ATGTTCATAAACGAATTTCCATTCATGGAG<br>Reverse<br>TCGCGTTCGTTCTGCACAGCTTCTTTTCGC
AgHNF4-dHLBD	XM_027986942.2	Forward<br>ATGTTCATAAACGAATTTCCATTCATGGAG<br>Reverse<br>TCGCGTTCGTTCTGCACAGCTTCTTTTCGC
dsGFP	LT726828.1	Forward<br>TAATACGACTCACTATAGGAATGGTGAGCAAGGGCGAGGA<br>Reverse<br>TAATACGACTCACTATAGGAGAAGTCGATGCCCTTCAGCT

dsHNF4	XM_027986942.2	Forward<br>TAATACGACTCACTATAGGGCAGGTACACTCGCCGCC<br>CAT<br>Reverse<br>TAATACGACTCACTATAGGGTTGCTCTTCGTAACTGGG<br>TC
*q- β-actin	KF018928.1	Forward<br>GTCTTCCCTTCCATCGTCGGCAGACCCCGT<br>Reverse<br>GCGGGTGTGTTGAAGGTTTCAAACATGATTTG
*q -EF1α	EU019874.1	Forward<br>GAAGCCTGGTATGGTTGTCGT<br>Reverse<br>GGGTGGGTTGTTCTTTGTG
*q -AgFAS	XM_050210906.1	Forward<br>ACGCTCAAGTGTATGCTA<br>Reverse<br>CGGATAGTATGTTGTTCAATA
*q -AgACC	XM_050207936.1	Forward<br>CTTCCGAGAATCCAGAATTACC<br><br>Reverse<br>CACCAAGAGCAAACATAGCCT
*q -AgSREBP	XM_027982255.2	Forward<br>AATGGCAGGAGCTTCACCAA<br>Reverse<br>CCGACTGTGCTTTGTTCACC
*q -AgLpR	XM_027994895.2	Forward<br>CACCGCAAATCACCAGCAAA<br>Reverse<br>GCTATGAGTCCGCTGTCCTC
*q -AgApoLpp	XM_050201488.1	Forward<br>TGCTGATTGCCCCCATCAAT<br>Reverse<br>CTGTATTTACCGTCCGGCGA

T7 promoter sequence was underlined. Additional information on PCR and qPCR is described in Materials and Methods. The Accession numbers for the genes are obtained through NCBI. *q represents the qPCR primers
